# Norovirus Gastroenteritis in a Birth Cohort in Southern India

**DOI:** 10.1371/journal.pone.0157007

**Published:** 2016-06-10

**Authors:** Vipin Kumar Menon, Santosh George, Rajiv Sarkar, Sidhartha Giri, Prasanna Samuel, Rosario Vivek, Anuradha Saravanabavan, Farzana Begum Liakath, Sasirekha Ramani, Miren Iturriza-Gomara, James J. Gray, David W. Brown, Mary K. Estes, Gagandeep Kang

**Affiliations:** 1 Department of Gastrointestinal Sciences, Christian Medical College, Vellore, India; 2 Department of Biostatistics, Christian Medical College, Vellore, India; 3 Virus Reference Department, Centre for Infection, Health Protection Agency, London, United Kingdom; 4 Department of Molecular Virology and Microbiology, Baylor College of Medicine, Houston, Texas, United States of America; Universidad Peruana Cayetano Heredia, PERU

## Abstract

**Background:**

Noroviruses are an important cause of gastroenteritis but little is known about disease and re-infection rates in community settings in Asia.

**Methods:**

Disease, re-infection rates, strain prevalence and genetic susceptibility to noroviruses were investigated in a birth cohort of 373 Indian children followed up for three years. Stool samples from 1856 diarrheal episodes and 147 vomiting only episodes were screened for norovirus by RT-PCR. Norovirus positivity was correlated with clinical data, secretor status and ABO blood group.

**Results:**

Of 1856 diarrheal episodes, 207 (11.2%) were associated with norovirus, of which 49(2.6%) were norovirus GI, 150(8.1%) norovirus GII, and 8 (0.4%) were mixed infections with both norovirus GI and GII. Of the 147 vomiting only episodes, 30 (20.4%) were positive for norovirus in stool, of which 7 (4.8%) were norovirus GI and 23 (15.6%) GII. At least a third of the children developed norovirus associated diarrhea, with the first episode at a median age of 5 and 8 months for norovirus GI and GII, respectively. Norovirus GI.3 and GII.4 were the predominant genotypes (40.3% and 53.0%) with strain diversity and change in the predominant sub-cluster over time observed among GII viruses. A second episode of norovirus gastroenteritis was documented in 44/174 (25.3%) ever-infected children. Children with the G428A homozygous mutation for inactivation of the *FUT2* enzyme (se^428^se^428^) were at a significantly lower risk (48/190) of infection with norovirus (*p* = 0.01).

**Conclusions:**

This is the first report of norovirus documenting disease, re-infection and genetic susceptibility in an Asian birth cohort. The high incidence and apparent lack of genogroupII specific immunity indicate the need for careful studies on further characterization of strains, asymptomatic infection and shedding and immune response to further our understanding of norovirus infection and disease.

## Introduction

Noroviruses are a leading cause of non-bacterial acute gastroenteritis (AGE) outbreaks in all age groups worldwide and are increasingly recognized as the second most common cause of sporadic AGE in children after rotavirus [1–3]. Noroviruses are non-enveloped, single-stranded positive-sense RNA viruses belonging to the family *Caliciviridae*. The viral genome consists of three open reading frames (ORFs), with ORF 1 encoding six non-structural proteins including the RNA dependent RNA polymerase (RdRp). ORF 2 encodes for the major capsid protein (VP1) and ORF 3 encodes a minor capsid protein (VP2) [4].

Noroviruses are divided into 5 genogroups (GI-GV) of which GI, GII and GIV are known to cause gastroenteritis in humans [5, 6]. Each genogroup is further divided into genetic clusters: 14 clusters in GI, 17 clusters in GII and 1 cluster in GIV have been described [7]. Among norovirus genogroups known to infect humans, GII noroviruses are the major genogroup causing outbreaks and sporadic infections worldwide and also predominate in community studies [8, 9].

Recent reports on norovirus infections from India were hospital-based studies of acute gastroenteritis [10–12], similar to the majority of reports in children from other parts of the world. There are limited data on norovirus infections in the community mainly because of the difficulty in conducting such studies, whether cross-sectional or longitudinal. Very recently, a Peruvian birth cohort study found that 71% of 189 children followed upto 2 years of age, experienced norovirus associated diarrhea, that genogroup II infections were common and that repeat infections by the same genogroup were common, but repeat infections by the same genotype were rare[13].We have previously reported a limited comparison of pediatric norovirus disease and strain distribution between children with diarrhea in the hospital and in this cohort and asymptomatic children from this cohort that demonstrated the importance of noroviruses as gastrointestinal pathogens in Indian children, the predominance of GII noroviruses and a low prevalence of GI [9].

Some studies have examined the course of norovirus infection, susceptibility and disease in healthy adults through volunteer and outbreak studies, but few are population-based estimates of norovirus disease burden in a community [8, 13]. The present study provides disease incidence and re-infection rates of norovirus genogroupsI and II, with evaluation of putative host markers of susceptibility in an Asian population followed from birth to the age of 3 years.

## Materials and Methods

### Study Design

All 1856 diarrheal stool samples and 147 available stool samples from the 155 vomiting only episodes (collected within 0–15 days of a vomiting episode) from a birth cohort of 373 children, followed up to 3 years were screened for the presence of GI and GII norovirus. The enrollment criteria and methods for follow up of the cohort, which was established to study rotavirus infections, included biweekly home visits, fortnightly surveillance stool sampling, monthly anthropometry (weight and length/height) measurements, in addition to collection of samples and clinical information for all diarrheal episodes [14, 15]. At least one fecal sample was collected in 98% of diarrheal episodes within one week of onset. Venous blood or saliva was collected from the children at the end of the study for genetic analysis. Household hygiene was assessed on a 24-point scale, using a structured questionnaire covering aspects of water, food and personal hygiene; this questionnaire has previously been validated and used in the same community[16]. Written informed consent was obtained from parents of all children prior to the enrollment, and the study was approved by the Institutional Review Board of the Christian Medical College, Vellore.

### Definition, assessment of severity and etiology of diarrhea

Diarrhea was defined as the passage of ≥3 loose watery stools during a 24‐hr period or in breastfed children alone, a change in the number or consistency of stools as reported by the mother. An episode was defined as at least one day of diarrhea, preceded and followed by ≥2 days without diarrhea. The severity of diarrhea was assessed using the Vesikari scoring system [17] that was developed for rotavirus but has been used to score severity of norovirus diarrhea in recent studies [8, 11, 18]. An episode was considered mild for a score ≤5, moderately severe for a score of 6–10 and severe for scores >10. Diarrheal stool samples were screened for parasite ova and cysts by microscopy, for bacterial pathogens by culture, and for rotavirus by enzyme-linked immunosorbent assay (Rota IDEIA; Dako, Ely, United Kingdom).

### RNA extraction and cDNA synthesis

Viral RNA was extracted from 20% fecal suspension in Minimal Essential Medium using guanidium isothiocyanate and silica [19].The RNA was eluted into 50μl of diethylpyrocarbonate treated water containing 40 units of RNase inhibitor (Invitrogen, Life Technologies, Paisley, UK). Complementary DNA (cDNA) was generated by reverse transcription in the presence of random primers (hexamers) [Pharmacia Biotech, United Kingdom] using Moloney murine leukemia virus reverse transcriptase (Invitrogen, Life Technologies, Paisley, UK).

### PCR for Norovirus GI

Screening of all stool samples from diarrheal episodes and vomiting only episodes for NoV GI was carried out using primers and conditions previously published [20]. The primers amplify an 84 bp fragment of the ORF 1–2 regions. Samples that were positive were cloned into the TOPO TA cloning kit as per the manufacturer’s instructions (Invitrogen, Life Technologies, UK). Clones were purified using QIAprep Spin Miniprep Kit (Qiagen, Valencia, USA) and subjected to automated sequence analysis.

### PCR for Norovirus GII

Two separate PCR reactions were carried out for all the samples using previously published primers specific to the RNA dependent RNA polymerase (RdRp) and ORF 1–2 regions as per previously published cycling conditions, amplifying a 113bp and 468bp fragment, respectively[20, 21]. Samples with amplification using either primer set were considered positive for norovirus and sequencing was attempted for all amplicons.

### Sequence Analysis

Sequencing of the positive amplicons was carried out by using the ABI PRISM Big Dye Terminator cycle sequencing ready reaction kit (Applied Biosystems, CA, USA). The amplicons corresponding to the ORF 1–2 junction region were sequenced when available; else the RdRp region was sequenced. Sequences were resolved using an automated DNA sequencer ABI PRISM 310 Genetic Analyzer. The sequences were imported into BioEdit software (version 7). Phylogenetic analysis was carried out using reference sequences from the norovirus molecular epidemiology database (Noronet) at www.rivm.nl and sequences from GenBank. Variants of NoV GII.4 were detected using the norovirus genotyping tool from http://www.rivm.nl/mpf/norovirus/typingtool. Neighbor joining method (10000 pseudo replicates) with MEGA software (version 4) was used to assign the samples to a genotype based on >90% homology at the nucleotide level with other strains within a given genotype [22].

### *Fucosyltransferase 2* (*FUT2*) genotyping for Secretor Status

Genomic DNA was isolated from buccal epithelial cells or blood using the QIAamp DNA Mini Kit (Qiagen, Hilden, Germany) according to manufacturer’s instructions. The amplification of a region of the *FUT2* gene and genotyping for two single nucleotide polymorphisms (SNPs) was carried out using published methods [23, 24]. The TaqMan Genotyping Master Mix (Applied Biosystems) and Custom TaqMan SNP Genotyping Assay for the 428 and 385 SNP of the *FUT2* gene designed by J. Le Pendu, France and M. Lay, USA (Applied Biosystems) respectively were used in a final volume of 20 μl.

### ABO and secretor phenotyping from saliva

Saliva samples that were boiled and cooled were used to coat 96-well high-binding plates (Costar, Corning, N.Y.) at room temperature for 4hrs and then blocked with 10% non-fat milk (blotto) overnight at 4°C. After washing, primary antibodies Anti-Le^a^ Gamma-clone (1:100), Anti-A Gamma-clone (1:200) and Anti-B Gamma-clone (1:100) from ImmucorGamma, Georgia, USA, BG-6 (anti-Le^b^) antibody (1:100) from Covance, Texas, USA were added and plates incubated at 37°C for 1hr. This was followed by the addition of a Goat anti-Mouse IgG-HRP (Sigma, USA) and UEA-1 lectin-HRP (Sigma, USA) at 37°C for 1 hr. The detection was carried out with TMB peroxidase substrate (KPL, Gaithersburg, MD) and the plates were read at OD450[25]. A pooled saliva sample and PBS were used as positive and negative controls, respectively.

### Statistical Analysis

The data were analyzed using STATA 10.0 for Windows (STATA Corp., TX, USA). Descriptive analysis was performed for all explanatory variables. Statistical significance of the observed differences in outcome between explanatory variables was assessed using Chi-square test or Fisher’s exact test for categorical variables and two-tailed *t*-test or Mann-Whitney U test for continuous variables, depending on the distribution of the data. A *p*-value of <0.05 was considered statistically significant. Seasonality of norovirus-associated diarrhea was assessed by fitting a sine curve to a time-series of the monthly stool positivity rates (proportion of diarrheal episodes attributed to norovirus divided by the total number of diarrheal episodes). The intensity of the seasonal fluctuation was computed by calculating the peak-to-low ratio using a modified version of the Edward’s technique [26].Association between distribution of Histo-Blood Group Antigen and secretor status among the norovirus infected and non-infected individuals were ascertained through logistic regression analysis and odds ratios (ORs) with 95% confidence intervals (CI) calculated.

Height-for age (HAZ), weight-for-height (WHZ) and weight-for-age (WAZ) z-scores were calculated using the 2006 WHO child growth standards as the reference population [27], and children classified as stunted (HAZ<-2 SD), wasted (WHZ<-2 SD), underweight (WAZ <-2 SD) or normal based on their respective z-scores.

## Results

A total of 1,856 episodes of diarrhea were experienced by the 373 children in the cohort, with a previously published incidence of gastrointestinal illness of 3.6 per child year in infancy and 1.64 and 1.16 per child year, respectively during the subsequent years [14]. The median (IQR) age of the first diarrheal episode was 3 (1–6) months. A total of 149 (149/373, 40%) children had 207 episodes of diarrhea associated with norovirus detection (details available in S1 Dataset). Norovirus GI and GII were detected in 49episodes (49/1856, 2.6%) and 150 episodes (150/1856, 8.1%) of diarrhea respectively; eight (8/1856, 0.4%) episodes were mixed infections with both norovirus GI and GII. Of the available 147 stool samples from the 155 vomiting only episodes which were collected within 0–15 days of vomiting, norovirus GI and GII were associated with 7 episodes (7/147, 4.8%) and 23 episodes (23/147, 15.6%) respectively, and there were no mixed infections.

Table 1 compares the clinical characteristics of diarrheal episodes associated with norovirus between GI and GII. The median (IQR) age at first symptomatic norovirus infection was 5 (3–7) months and 8 (5–15) months among norovirus GI and GII respectively. The median (IQR) duration of a diarrheal episode and proportion associated with vomiting and fever were similar for the two viral genogroups (Table 1).

**Table 1:** Comparison of clinical characteristics between norovirus GI and GII diarrheal episodes.

	Variable	NoV GI	NoV GII	*p*-value
Overall	Number of episodes^**a**^	49	150	—
	Median (IQR) duration (in days)	3 (2–4)	3 (2–5)	0.542^**b**^
	Median (IQR) Vesikari score	5 (5–8)	5 (5–7)	0.749^**b**^
	Number (%) of episodes with associated vomiting	12 (24.5)	33 (22.8)	0.804^**c**^
	Number (%) of episodes with associated fever	14 (28.6)	33 (22.8)	0.443^**c**^
Before introduction of supplementary feeding	Number of episodes^**a**^	8	11	**—**
	Median (IQR) duration(in days)	4 (3–5.5)	3 (2–5)	0.399^**b**^
	Median (IQR) Vesikari score	5 (4.5–6)	5 (5–7)	0.465^**b**^
	Number (%) of episodes with associated vomiting	0 (0%)	3 (27.3)	0.228^**d**^
	Number (%) of episodes with associated fever	1 (12.5)	2 (18.2)	1.000^**d**^
After introduction of supplementary feeding	Number of episodes^**a**^	41	134	**—**
	Median (IQR) duration(in days)	3 (2–4)	3 (2–5)	0.848^**b**^
	Median (IQR) Vesikari score	6 (5–8)	5 (5–7)	0.475^**b**^
	Number (%) of episodes with associated vomiting	12 (29.3)	30 (22.4)	0.367^**c**^
	Number (%) of episodes with associated fever	13 (31.7)	31 (23.1)	0.268^**c**^
During infancy	Number of episodes^**a**^	42	86	**—**
	Median (IQR) duration(in days)	3 (2–4)	3 (2–5)	0.630^**b**^
	Median (IQR) Vesikari score	5 (5–8)	6 (5–9)	0.291^**b**^
	Number (%) of episodes with associated vomiting	9 (21.4)	23 (26.7)	0.514^**c**^
	Number (%) of episodes with associated fever	11 (26.2)	22 (25.6)	0.941^**c**^
Post infancy (2^nd^ and 3^rd^ year of life)	Number of episodes	7	59	**—**
	Median (IQR) duration (in days)	3 (2–5)	2 (2–3)	0.229^**b**^
	Median (IQR) Vesikari score	7 (6–8)	5 (4–7)	0.023^**b**^
	Number (%) of episodes with associated vomiting	3 (42.9)	10 (16.9)	0.131^**d**^
	Number (%) of episodes with associated fever	3 (42.9)	11 (18.6)	0.159^**d**^

a = Includes both primary and secondary infections

b = Mann-Whitney U test

c = Chi-square (χ2) test

d = Fisher’s exact test

### Re-infection

One hundred and thirty of the 149 (87.2%) children who ever had a norovirus associated diarrhea had only one episode during the 3-year follow-up period. Forty-four of 149 (29.5%) children had a re-infection with either genogroup. Children showed a higher rate of re-infection with norovirus GII (30/123, 24.3%) compared to norovirus GI (6/51, 11.8%). Eight of the 174 children (4.6%) had both norovirus GI and GII infections. Among the 30 children with 2 or more norovirus GII positive diarrheal episodes, three children experienced the second episode 6, 8 and 9 days after the first episode, which may represent continued shedding.

**Fig 1:**
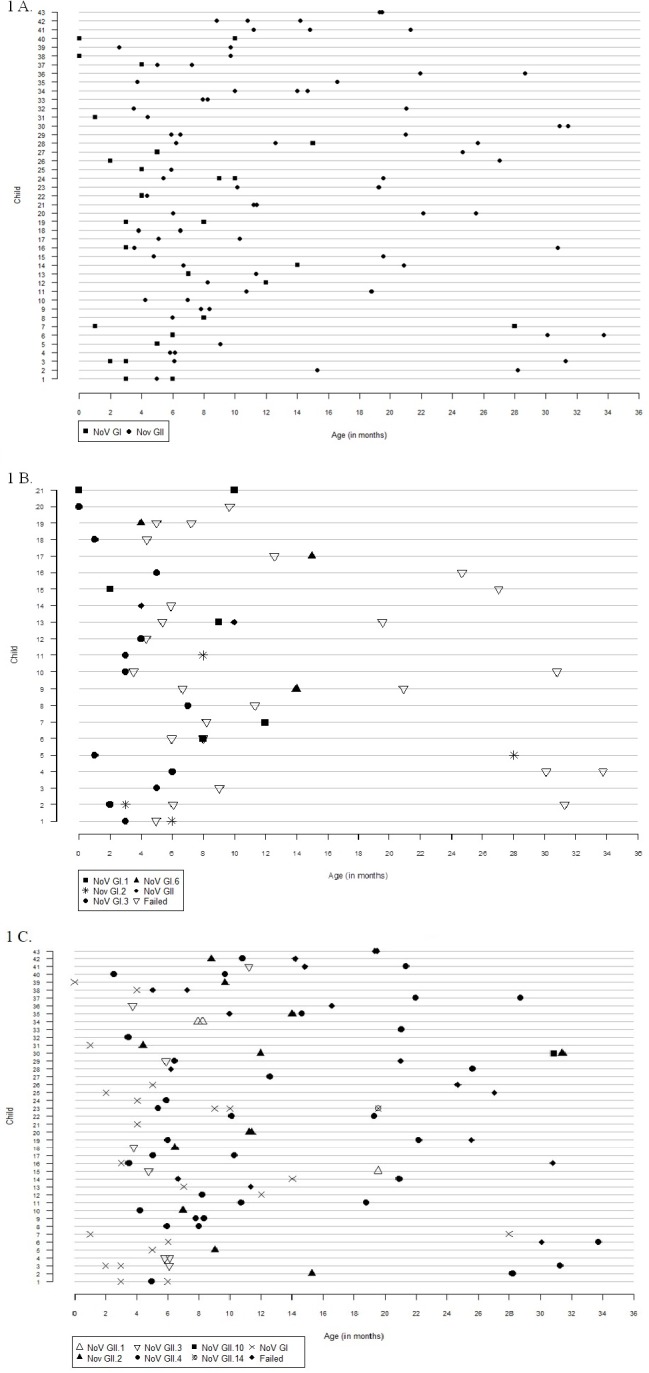
Norovirus genotypes in primary and subsequent diarrheal episodes in children who had more than one infection. Each line represents one child with re-infection, while the age in months is on the x- axis. Norovirus genotypes are represented by symbols. Samples that failed sequencing have been denoted as Failed in the figure. A) Children with re-infections with either norovirus GI and GII. B) All norovirus GI infection children who were re-infected with either GI or GII noroviruses. GI genotypes are shown, while GII infections are shown only as genogroup. C) All norovirus GII infection children who were re-infected with either GI or GII noroviruses. GII genotypes are shown while GI infections are shown only as genogroup.

Only one child (1.9%) with norovirus GI re-infection was re-infected with the same genotype and the rest had a different genotype during re-infection (Fig 1). The duration between primary and re-infection with the same genotype was 270 days. The shortest and longest duration between re-infection with a different genotype was 26 and 811 days.

Of the 30 children with norovirus GII re-infection, genotype could not be identified for 7 samples. Of the remaining, 12 children were re-infected with the same genotype, three of whom may have had continuous shedding as stated above, and 11 with a different genotype (Fig 1). Reinfections with GII.4 in six of nine re-infected children were with different variants (Table 2). The shortest and longest duration between re-infection with the same genotype, other than the three who were less than 10 days, were 74 and 534 days. Re-infection with different genotypes ranged from 16 days to 767 days.

**Table 2:** Distribution of norovirus variants of GII.4.

Child No.*	Sample No.	ORF2	ORF2_variant	ORF2_variant
8	CRI6134	GII.4	Hunter_2004	II.4|2004
	CRI8497	GII.4	Hunter_2004	II.4|2004
9	CRI3819	GII.4	Lanzou_2002	II.4|2002CN
	CRI4419	GII.4	Lanzou_2002	II.4|2002CN
11	CRI7807	GII.4	US95_96	II.4|1995
	CRI16705	GII.4	Hunter_2004	II.4|2004
17	CRI5799	GII.4	Lanzou_2002	II.4|2002CN
	CRI11124	GII.4	Hunter_2004	II.4|2004
19	CRI14276	GII.4	Kaiso_2003	II.4|2003
	CRI30481	GII.4	Asia_2003	II.4|2005
22	CRI19650	GII.4	Hunter_2004	II.4|2004
	CRI28799	GII.4	Asia_2003	II.4|2005
32	CRI2563	GII.4	Lanzou_2002	II.4|2002CN
	CRI19884	GII.4	Hunter_2004	II.4|2004
37	CRI24530	GII.4	Hunter_2004	II.4|2004
	CRI31001	GII.4	Hunter_2004	II.4|2004
40	CRI9506	GII.4	Kaiso_2003	II.4|2003
	CRI17383	GII.4	Hunter_2004	II.4|2004

*Child number denotes the child in the Fig 1C.

### Presence of co-pathogens

Mixed infections with other pathogens were seen in 56/207 (27%) norovirus-associated diarrheal episodes, of which 52 episodes had a single co-pathogen and 4 had two co-pathogens. The commonest co-pathogen isolated was rotavirus, which was present in 36/56(64.2%) episodes of mixed infections, followed by *Giardia* in 9/56 (16.1%) episodes; other co-pathogens were: *Cryptosporidium* (5/56, 8.9%), *Aeromonas* (4/56, 7.1%), *Vibrio* (3/56, 5.4%), *Shigella* (2/56, 3.6%) and *Ascaris* (1/56, 1.7%). The comparison of clinical features between diarrheal episodes of single and mixed infections is presented in Table 3. Episodes of mixed infection had higher association with comorbidities such as vomiting and fever, and were more severe.

**Table 3:** Comparison of clinical features between diarrheal episodes of single infections with norovirus and mixed infections of norovirus and other enteric pathogens.

	Norovirus GI			Norovirus GII		
Clinical features	Single infection(N = 47)	Mixed infection(N = 9)^a^	*p*-value	Single infection (N = 105)	Mixed infection(N = 47)^b^	*p*-value
Number (%) of episodes with associated vomiting	9 (19.2)	4 (44.4)	0.189^**c**^	18 (17.1)	16 (34.0)	0.021^**e**^
Number (%) of episodes with associated fever	15 (36.2)	1 (11.1)	0.421^**c**^	18 (17.1)	17 (36.2)	0.010^**e**^
Number (%) of episodes with associated dehydration	5 (10.6)	1 (11.1)	1.000^**c**^	7 (6.7)	6 (13.0)	0.205^**e**^
Median (IQR) Vesikari score	5 (5–8)	7 (5–8)	0.356^**d**^	5 (5–7)	6 (5–9)	0.045^**d**^

a = Co-pathogens isolated: Rotavirus, *Giardia*, *Aeromonas*

b = Co-pathogens isolated: Rotavirus, *Giardia*, *Cryptosporidium*, *Vibrio*, *Shigella*,*Aeromonas*, *Ascaris*

c = Fisher’s exact test

d = Mann-Whitney U test

e = Chi-square (χ2) test

### Seasonality of norovirus-associated diarrhea

**Fig 2:**
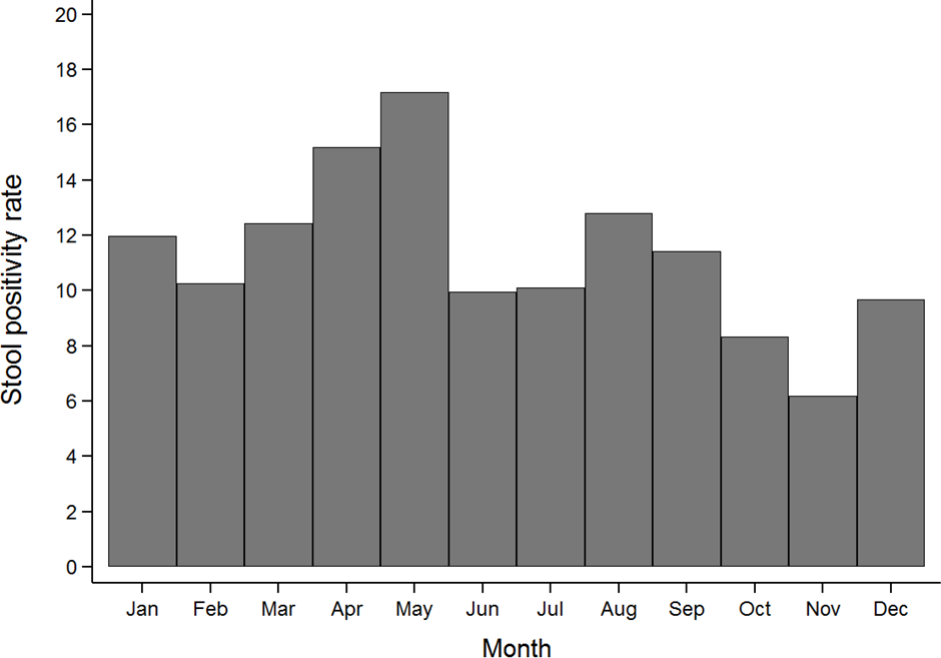
Seasonal fluctuations in the monthly occurrence of episodes of norovirus-associated diarrhea. The y-axis represents the monthly stool positivity rates (proportion of diarrheal episodes attributed to norovirus divided by the total number of diarrheal episodes). The x-axis represents the month of the year.

Norovirus-associated diarrhea was seen to occur during all months of the year, ranging from a stool positivity rate of 6.2% in November to 17.2% in May (Fig 2). The peak-to-low (95% CI) ratio of seasonal intensity of occurrence of norovirus diarrhea was 1.57 (1.05–2.20), suggestive of a mild increase in the proportion of norovirus-associated diarrheal episodes during the summer season.

### Age and norovirus-associated diarrhea

A total of 50 (87.7%) and 98 (62%) episodes of diarrhea in children ≤1 year of age were associated with norovirus GI and GII respectively. The median age of norovirus diarrhea was 7 (4.5–12) months. Median age (IQR) of diarrhea with norovirus GI and GII were 5 (3–7) months and 8.8(5.7–15.3) months respectively. The median (IQR) duration in infants was similar between the two genogroups of the virus (Table 1). Diarrheal severity was greater in norovirus GII episodes occurring during infancy (Median [IQR] Vesikari score = 6 [5–9]) than at a later age (Median [IQR] Vesikari score = 5 [4–7], *p* = 0.016); this was reversed for norovirus GI episodes, although the difference was not statistically significant (Median [IQR] Vesikari score = 5 [5–7] vs. 7 [6–8], *p* = 0.111). No difference in associated vomiting or fever between episodes occurring below and above the age of 1 year was observed among the two viral genogroups.

The overall incidence of norovirus diarrhea was 0.14 (0.12–0.16) per child-year which is lower than the incidence of rotavirus diarrhea in the same population, 0.25 (0.22–0.29) per child-year (Gladstone et al., personal communication). The incidence of norovirus diarrhea was highest during the first year of life, 0.26 (0.22–0.32) per child-year and decreased to 0.09 (0.07–0.13) and 0.07 (0.05–0.10) per child-year during the second and third year, respectively.

### Breastfeeding and norovirus-associated diarrhea

Eight (8/57, 14%) GI and 11 (11/158,7%) GII associated diarrheal episodes occurred before the introduction of supplementary feeding or weaning (Table 1). No significant patterns were observed in the duration (IQR) of breastfeeding among the norovirus infected (n = 149, 3 [1–4] months) and uninfected children (n = 224, 2 [1–3] months); *p* = 0.753. There was no difference in the severity of diarrheal episodes, vomiting or febrile symptoms pre- and post-weaning in children with either genogroup.

### Household hygiene and norovirus-associated diarrhea

The median (IQR) household hygiene score among the norovirus infected and uninfected children was 11 (8–14) and 11 (9–13.5), respectively (*p* = 0.500). When the household hygiene scores were compared between children with single (n = 105, 11 [8–13]) and multiple (n = 44, 10 [7–14]) norovirus infections, they were found to be similar (*p* = 0.730). Similarly, children with only GI infections (n = 26, 10[8–13]) had comparable hygiene scores to children with only GII (n = 98, 11 [8–14],*p* = 0.758) infections.

### Growth and norovirus-associated diarrhea

**Fig 3:**
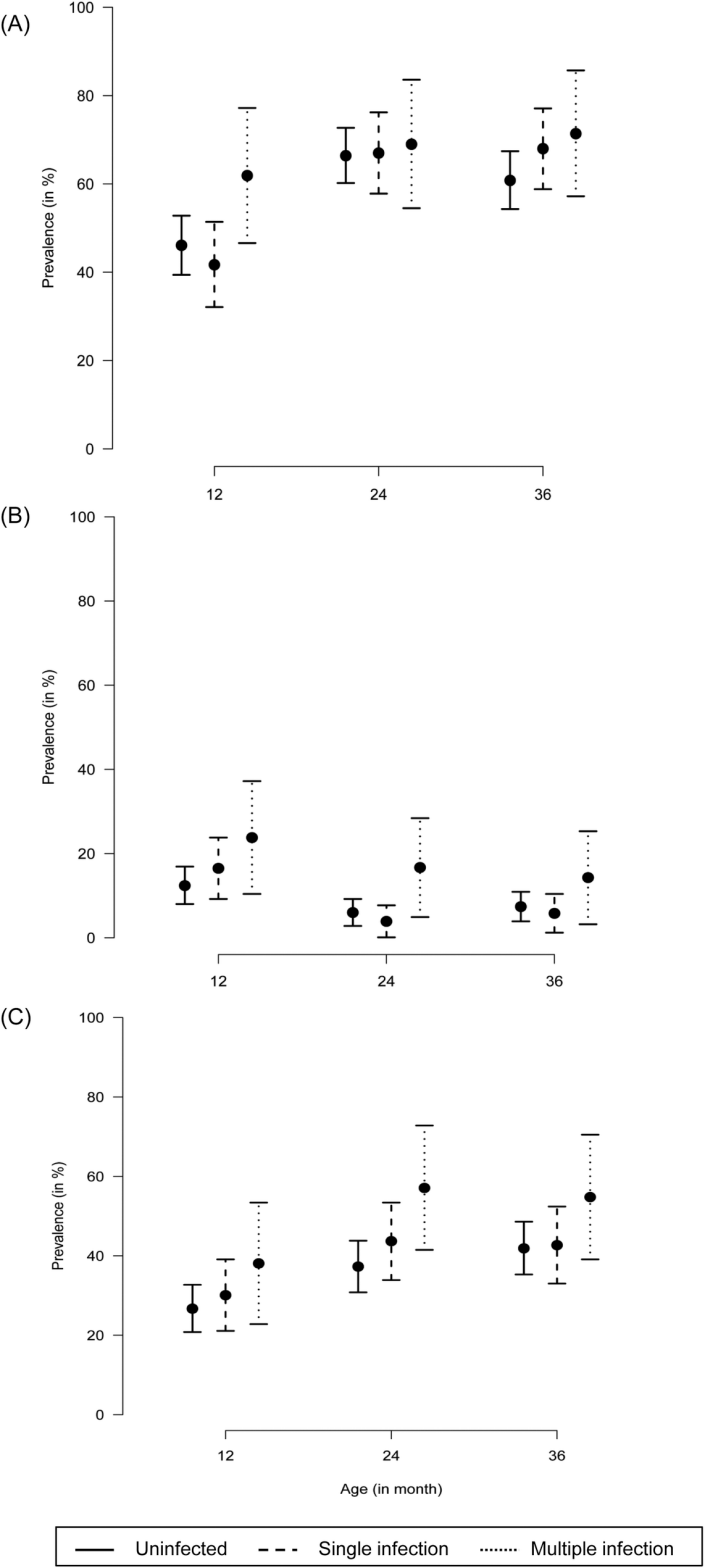
**Proportion (with 95% CI) of (A) stunted, (B) wasted and (C) underweight children at 12, 24 and 36 months of age.** The y-axis depicts the proportion of children with growth deficiency whereas the x-axis represents age in months. The point prevalence is presented as a circle and the 95% CI is represented as vertical lines. The solid vertical line represents uninfected children, the dashed and dotted lines represent those with single and multiple norovirus infections, respectively.

Complete anthropometry data was available for 362 (97.1%) of the 373 children under observation. The proportion (with 95% CI) of stunted, wasted and underweight children at 12, 24, and 36months of age among those with and without norovirus infection is presented in Fig 3. The proportions of stunted, wasted and underweight children were comparable between those with and without norovirus infection at all the three time points. However, when the anthropometric data were compared between children with single and multiple norovirus infections, a higher proportion of children with multiple infections were stunted (61.9 vs. 41.7%, *p* = 0.027) and wasted (16.7% vs. 3.9%, *p* = 0.014) at 12 and 24 months of age, respectively (Fig 3). The proportion of stunted, wasted and underweight children was comparable between children with norovirus GI and GII infections.

### Genotyping

A total of 215 samples were genotyped by sequencing of the amplicons. Three GI and 26 GII samples failed in sequencing reactions, but are included in the overall analysis because all PCRs were repeated at least twice, and band sizes re-confirmed with absence of non-specific bands.

**Fig 4:**
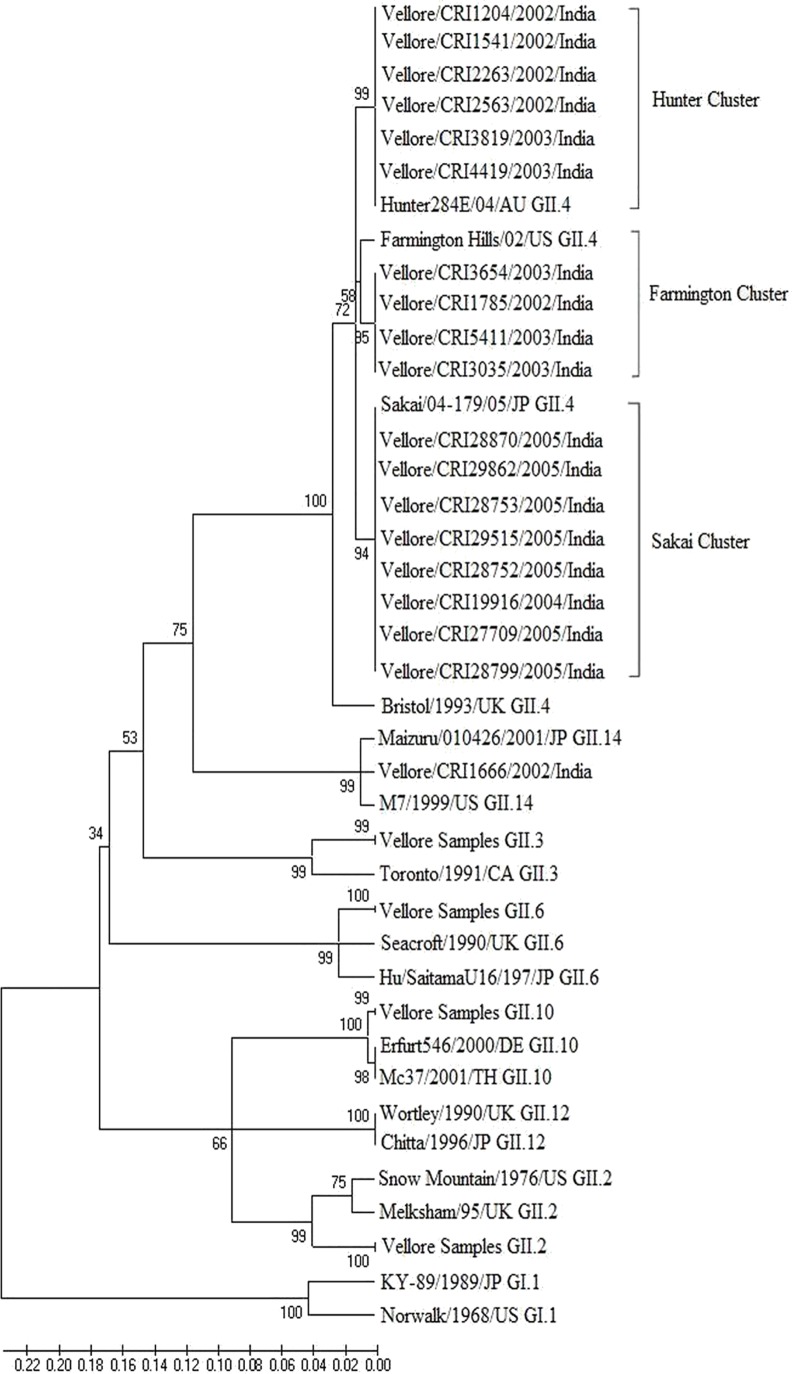
Dendrogram constructed using neighbor joining of nucleotide sequences corresponding to fragment of 468 bp of the ORF 1–2 region of the Norovirus capsid encoding gene. Bootstrap values for 10000 pseudo replicates are shown. The reference strains included for comparison with accession numbers are: Hunter284/04/AU (DQ078794), Farmington Hills/02/US (AY502023), Sakai/04-179/05/JP (AB220922), Bristol/1993/UK (X76716), Maizuru/010426/2001/JP (EF547404), M7/1999/US (AY130761), Toronto/1991/CA (AAA18930), Seacroft/1990/UK (AJ277620), Hu/SaitamaU16/197/JP (AB039778), Erfurt546/2000/DE (AF427118), Mc37/2001/TH (AY237415), Wortley/1990/UK (AJ277618), Chitta/1996/JP (AB032758), Snow Mountain/1976/US (AY134748), Melksham/95/UK (X81879), KY-89/1989/JP (L23828), Norwalk/1968/US (M87661).

Sequence analysis of the GI genogroups showed a predominance of GI.3 genotype in 23/57 (40.3%) of the samples, followed by GI.1 (15/57, 26.3%), GI.2 (9/57, 15.7%) and GI.6 (6/57, 10.5%). Phylogenetic analysis of norovirus GII genogroup of either the capsid or the RdRp region (Fig 4) showed the predominance of GII.4 genotype in 70/132 (53.0%) of the samples, followed by GII.2 (28/132, 21.2%) and GII.3 (18/132,13.6%), respectively.

The GII.4 strains were placed in 3 sub-clusters when analyzed with reference sequences. Of the 35 samples identified as GII.4 at the ORF 1–2 capsid junctions, 17 strains with 96% nucleotide identity clustered in the Hunter sub-cluster, 6 with 93% nucleotide identity with the Farmington sub-cluster and 12 with 98% nucleotide identity with the Sakai sub-cluster. Fifteen children between the ages of 2.5–8.3 months were infected with the Hunter sub-cluster, with 2 children having two episodes of infection 17 and 159 days apart, respectively. Six children between the ages of 4.2–8.6 months were infected with the Farmington sub-cluster. The Hunter and Farmington sub-cluster infections were seen in children between the years 2002–2003, indicating the circulation of this sub-cluster in the community. However, there was a predominance of the Sakai sub-cluster in 12 children who were infected between 2004–2005. Nine samples were genotyped as GII.3 and clustered with 94% nucleotide identity to the Toronto strain. Ten samples were genotyped as GII.2 with 98% nucleotide identity to the Snow Mountain strain.

Nucleotide sequences of positive samples from the study have been deposited in the GenBank and the accession numbers are JN654720-JN654766.

### Association of blood group and secretor status with norovirus infection

ABO blood group and secretor status were determined for 293 children from whom appropriate samples were available. The most common ABO blood group was O (39.3%), followed by B (32.5%), A (20.9%) and AB (7.2%). One hundred eighty-six children (186/305, 60.9%) were secretor positive.

Secretor status was significantly associated with overall norovirus GI (Table 4), but not with norovirus GII infection (Table 5). No significant association between the ABO blood group and norovirus GI or GII infection was observed (Tables 4 and 5). Among the 48 children with GI infection, those with GI.1 genotype (n = 15) were all secretor positive, whereas among the children with non GI.1 infection (n = 33), 84.9% were secretor positives although the difference was not statistically significant (*p* = 0.167). For the 115 children with GII infection, 75% of those with GII.4 (n = 56) were secretor positives, whereas only 57.8% of children with non GII.4 infection (n = 57) were secretor positives (*p* = 0.054). Moreover, genotyping for the *FUT2* SNP showed that children with the G428A homozygous mutation for inactivation of the *FUT2* enzyme (se^428^se^428^) are significantly at a lower risk of infection with norovirus (Tables 4 and 5). There was no significant association between norovirus diarrhea with any genotype with any specific blood group.

**Table 4:** Distribution of Histo-Blood Group and Secretor status among norovirus GI infected and non-infected individuals.

	Norovirus GI infected (n = 48)	Norovirus GI negative (n = 257)	OR	95% CI	*p*- value
**Blood Group**					
O	22 (46%)	98 (38%)	1.57	(0.66–3.76)	0.31
B	15 (31%)	84 (33%)	1.25	(0.50–3.14)	0.64
AB	3 (6%)	19 (7%)	1.11	(0.27–4.60)	0.89
A	8 (17%)	56 (22%)	1		
**Secretor Phenotype**					
Secretor	43 (90%)	157 (61%)	5.48	(2.09–14.29)	0.001
Non-Secretor	5 (10%)	100 (39%)	1		
**Genotyping for FUT2 SNP (428G>A)**					
Se^428^Se^428^	23 (48%)	130 (51%)	1		
Se^428^se^428^	13 (27%)	67 (26%)	1.22	(0.62–2.39)	0.56
se^428^se^428^	12 (25%)	60 (23%)	0.32	(0.11–0.96)	0.04
**Genotyping for FUT2 SNP (385A>T)**					
Se^385^Se^385^	21 (44%)	72 (28%)	1		
Se^385^se^385^	20 (42%)	96 (37%)	0.71	(0.36–1.42)	0.34
se^385^se^385^	7 (14%)	89 (35%)	0.27	(0.11–0.67)	0.01

SeSe: Homozygous wildtype for *FUT2;* Sese: Heterozygous for inactivation of *FUT2;*sese: Homozygous for inactivation of *FUT2*

**Table 5:** Distribution of Histo-Blood Group and Secretor status among norovirus GII infected and non-infected individuals.

	Norovirus GII infected (n = 115)	Norovirus GII negative (n = 190)	OR	95% CI	*p*-value
**Blood Group**					
O	43 (37%)	77 (41%)	0.93	(0.50–1.75)	0.82
B	38 (33%)	61 (32%)	1.04	(0.54–1.99)	0.91
AB	10 (9%)	12 (6%)	1.39	(0.52–3.70)	0.51
A	24 (21%)	40 (21%)	1		
**Secretor Phenotype**					
Secretor	77 (67%)	123 (65%)	1.10	(0.68–1.80)	0.69
Non-Secretor	38 (33%)	67 (35%)	1		
**Genotyping for FUT2 SNP (428G>A)**					
Se^428^Se^428^	68 (59%)	89 (47%)	1		
Se^428^se^428^	31 (27%)	53 (28%)	0.77	(0.44–1.32)	0.34
se^428^se^428^	16 (14%)	48 (26%)	0.44	(0.23–0.83)	0.01
**Genotyping for FUT2 SNP (385A>T)**					
Se^385^Se^385^	33 (29%)	60 (32%)	1		
Se^385^se^385^	44 (38%)	72 (38%)	1.11	(0.63–1.96)	0.72
se^385^se^385^	38 (33%)	58 (31%)	1.19	(0.66–2.15)	0.56

SeSe: Homozygous wildtype for *FUT2;* Sese: Heterozygous for inactivation of *FUT2;* sese: Homozygous for inactivation of *FUT2*

## Discussion

This is the first longitudinal study of norovirus gastroenteritis in a birth cohort of children in India. Most estimates of disease burden are based on hospital studies, which sample the more severe end of the spectrum of disease, and do not have community based denominator data. In this birth cohort from an urban slum in southern India, norovirus was a significant cause of diarrhea, associated with 11.2% of episodes, second only to rotavirus, and affecting almost half the cohort. In a recent study from India, 10.7% positivity was found in children <7 years of age, with 40% positivity in the ≤1 year olds [10], but this was a cross-sectional study in children presenting to hospital with gastroenteritis. Community based studies from developed countries like Europe have reported higher norovirus rates, 24.5% in children <5, and 28% in those ≤1 year old in the UK [28], and in the Netherlands, 14% in children <4 years and 14.3% in children ≤1 year of age [29], but these findings are in settings where higher rates of viral gastroenteritis are reported in children presenting to hospitals than in developing countries.

A majority of the norovirus reports from developing countries are hospital based and the detection ranges from 5.5% in Vietnam, 15% in Nicaragua, 9.3% in Tunisia, 30% in Iraq, 3.7% in Brazil and 32.1% in urban Peru, 11.3% in Malawi and 14% in Guatemala [30–37]. Two cohort studies carried from Chile and Peruvian Amazon have shown 18% and 25% of acute diarrhea episodes in children <24 months were due to norovirus respectively [38, 39]. A recently published birth cohort study among children from Peruvian urban community showed a prevalence of 22.8% among the diarrheal stool samples screened[13]. In our study, a significantly higher proportion of children with multiple infections were stunted, wasted and underweight at age 12 and 24 months irrespective of genotype which is in contrast to that seen among children from the Peruvian study where GII exhibited a more significant effect[13].

Children who had vomiting only episodes had high positivity for norovirus GI or GII (30/147, 20.4%) in stool compared to the proportion of diarrheal stools positive for norovirus (215/1856, 11.6%). Earlier studies on children have reported a higher percentage of vomiting episodes than diarrheal episodes in norovirus associated outbreaks of gastroenteritis, described as 'winter vomiting disease'[40].A study on norovirus gastroenteritis in military trainees from the USA also reported a higher norovirus positivity in vomiting episodes (80%) compared to diarrheal episodes (67%)[41].

The median Vesikari score for norovirus GI and GII diarrhea was 5, similar to studies from other developing countries [8, 42], and was less severe than that associated with rotavirus disease in this cohort, which had a median severity score of 7.6 [43]. The median age of the first symptomatic infection of norovirus GI and GII was 5 and 8 months respectively, slightly younger than 9 months and 11 months as reported from studies in Chile and Brazil, respectively [8, 44].In a study examining the global age distribution of pediatric norovirus cases, approximately 70% of norovirus cases occurred in the age group between 6 months to 2 years [45]. The study recommended completion of norovirus vaccination in children by 6 months of age to potentially prevent a greater burden of disease compared to completion of vaccination at 12 months. The recommendation would hold true for our study population where the median age (IQR) of norovirus diarrhea was 7 (4.5–12) months. Early completion of immunization is likely to have a greater benefit in reducing disease burden, particularly for GII infections where the primary infection was more severe.

The severity and frequency of vomiting and fever associated with norovirus was higher among the GII infected children (Table 1); this finding is similar to studies from Peru [13, 38].In human volunteer challenge studies and vaccine trials, the definition of norovirus illness includes individuals who experience only vomiting in the absence of diarrhea as a clinical endpoint [46–48]. In this study, the examination of stool samples from children who presented with vomiting alone as a primary clinical symptom suggests that there may be an underestimation of norovirus disease burden by exclusion of cases presenting with vomiting only.

Symptomatic norovirus associated diarrhea was detected in children during the first 6 months of age suggesting that breast feeding did not confer protection, unlike the absence of symptomatic infection among younger children in the Peruvian study [13]. Re-infection with norovirus was associated with diarrhea in a quarter of ever-infected children, this is in contrast with reports from the Chilean birth cohort where 40% (10/25) of re-infections with norovirus were symptomatic, the lower number of re-infections seen could be due to the difference in size of the study population [8]. In the birth cohort from Peru, repeat infections by the same genogroup of norovirus were seen, but the majority of the reinfections were by a different genotype or variant of GII.4. The GII.4 variants described in Peru were 2006b, 2007, 2008 and 2010 [13]. The data in this study showed higher rates of reinfections with the same genogroup and genotype of norovirus than in Peru. Since these studies were carried out in distinct time periods (Vellore, 2002–2006 and Peru, 2007–2011), some of these differences may be due to the difference in circulating GII.4 variants between the two studies (Table 2).Re-infection with the same genogroup and genotype was observed in this study suggesting that lower genogroup or genotype specific immunity may occur in India, in contrast to the Peruvian study where genotype-specific immunity was observed[13].

A high degree of genetic diversity was seen among circulating noroviruses, with four GI genotypes and nine genotypes of norovirus GII detected. These data are similar to other studies where a higher diversity of norovirus has been documented in gastroenteritis in the community when compared to outbreaks, generally in the adult and/or elderly population [4, 28, 49–53]. GI.3 (40.3%) was the predominant strain detected in the cohort followed by GI.1 (26.3%), GI.2 (15.7%) and GI.6 (10.5%). GII.4 was the predominant genotype while GII.2 (17.7%) and GII.3 (11.4%) were also detected in significant proportions, similar to previous reports [28, 49]. Other studies in India have reported a predominance of GII.4 strains; however, these were predominantly outbreaks studies [9, 10]. The GII.4 strains detected in the current study clustered into3sub-clusters, with a temporal distribution of the Hunter sub-cluster being replaced by Farmington and then by Sakai. Evolutionary analysis studies have shown that the newer strains originate by amino acid changes due to positive selection and recombination which drives the persistence of newer strains in the human population [54].

The link between the Histo-Blood Group Antigen (HBGA) binding and secretor status is well documented for the prototype GI virus, Norwalk virus. In this study, there was no significant association between blood groups and norovirus GI infection, but higher infection rates were seen among secretors compared to non-secretors, which is similar to previous studies of volunteer challenge and *in vitro* binding studies that have shown that carbohydrate binding is essential for GI infection [23, 55–57]. There was no significant association between blood groups or secretor status with norovirus GII infection which is consistent with other reports that have shown no association between host genetic factors and symptomatic infection of norovirus GII[58–60]. Expression of the *FUT2* gene and susceptibility to norovirus infection was examined by checking for two common mutations in Asian populations, which showed that children with a nonsense mutation at G428A were significantly at a lower risk of infection with norovirus, as has been previously shown by other studies [61, 62].

In summary, this report shows that norovirus infections are a common cause of pediatric gastroenteritis in southern India. A previous report from the same region found 15% of hospitalized gastroenteritis cases in children <5 years of age to be positive for norovirus [9]. In the community, norovirus infections were associated with mild disease, but symptomatic re-infections were common. The continued replacement of strains in the population suggests the need for strain specific vaccines, or identification of epitopes that are conserved across the norovirus variants could be used to generate broadly protective vaccines. In order to assess the true rate of infection with noroviruses in this community and the role of prior infections in protection from subsequent infection or disease, the investigation of asymptomatic shedding and immune responses would be of interest.

## Supporting Information

S1 Dataset Details of children with norovirus diarrhea. (XLSX)
